# Neurocranial Osteology and Neuroanatomy of a Late Cretaceous Titanosaurian Sauropod from Spain (*Ampelosaurus* sp.)

**DOI:** 10.1371/journal.pone.0054991

**Published:** 2013-01-23

**Authors:** Fabien Knoll, Ryan C. Ridgely, Francisco Ortega, Jose Luis Sanz, Lawrence M. Witmer

**Affiliations:** 1 Departamento de Paleobiología, Museo Nacional de Ciencias Naturales, Consejo Superior de Investigaciones Científicas, Madrid, Spain; 2 Department of Biomedical Sciences, Heritage College of Osteopathic Medicine, Ohio University, Athens, Ohio, United States of America; 3 Departamento de Física Matemática y de Fluidos, Facultad de Ciencias, Universidad Nacional de Educación a Distancia, Madrid, Spain; 4 Departamento de Biología, Facultad de Ciencias, Universidad Autónoma de Madrid, Madrid, Spain; Ludwig-Maximilians-Universität München, Germany

## Abstract

Titanosaurians were a flourishing group of sauropod dinosaurs during Cretaceous times. Fossils of titanosaurians have been found on all continents and their remains are abundant in a number of Late Cretaceous sites. Nonetheless, the cranial anatomy of titanosaurians is still very poorly known. The Spanish latest Cretaceous locality of “Lo Hueco” yielded a relatively well preserved, titanosaurian braincase, which shares a number of phylogenetically restricted characters with *Ampelosaurus atacis* from France such as a flat occipital region. However, it appears to differ from *A. atacis* in some traits such as the greater degree of dorsoventral compression and the presence of proatlas facets. The specimen is, therefore, provisionally identified as *Ampelosaurus* sp. It was CT scanned, and 3D renderings of the cranial endocast and inner-ear system were generated. Our investigation highlights that, although titanosaurs were derived sauropods with a successful evolutionary history, they present a remarkably modest level of paleoneurological organization. Compared with the condition in the basal titanosauriform *Giraffatitan brancai*, the labyrinth of *Ampelosaurus* sp. shows a reduced morphology. The latter feature is possibly related to a restricted range of head-turning movements.

## Introduction

In 2007, in the course of the construction of a high-speed rail track connecting Madrid with Valencia, an exceptional fossil site was discovered in the Villalba de la Sierra Formation at a locality named “Lo Hueco,” near the village of Fuentes, Castile-La Mancha, Spain. Over the course of several months, a large-scale emergency excavation allowed thousands of specimens of plants, invertebrates, and vertebrates of late Campanian-early Maastrichtian age to be saved [1]. Together with crocodiles, the sauropods represent the largest part of the biomass at Lo Hueco. The large number of sauropod elements from Lo Hueco (many of which are in articulation) are yet to be fully prepared and described, but preliminary observations suggest that more than one titanosaurian species is present. Among this rich sauropod collection, only a very limited number of cranial elements were collected: two braincases (likely to represent distinct taxa) and a number of isolated teeth. This is not surprising given the fragility of the skull in sauropods. The skull elements are extremely important remains as the cranial anatomy of titanosaurian sauropods is currently very poorly known, except for a few remarkable exceptions (see in particular [2]–[7]).

The aim of the present paper is to present a detailed osteological description as well as digital reconstructions of the endocast and endosseous labyrinth of the inner ear based on CT scanning of a significant sauropod cranial specimen from Lo Hueco: a well preserved, though somewhat incomplete, braincase.

### Repository Abbreviations

ANS, Academy of Natural Sciences, Philadelphia, PA, USA; FAM, Mairie de Fox-Amphoux, Fox-Amphoux, France; FGGUB: Facultatea de Geologie şi Geofizică a Universită ii din Bucureşti, Bucharest, Romania; GSI: Geological Survey of India, Kolkata, India; MCCM: Museo de las Ciencias de Castilla-La Mancha, Cuenca, Spain; MCNA: Museo de Ciencias Naturales de Álava, Vitoria, Spain; MDE: Musée des Dinosaures, Espéraza, France; MNHN: Muséum National d’Histoire Naturelle, Paris, France; PIN: Paleontologicheskii Institut, Rossiiskaya Akademiya Nauk, Moscow, Russia; TMM: Texas Memorial Museum, Austin, TX, USA; Z. PAL: Instytut Paleobiologii, Polska Akademia Nauk, Warsaw, Poland.

## Materials and Methods

**Figure 1:**
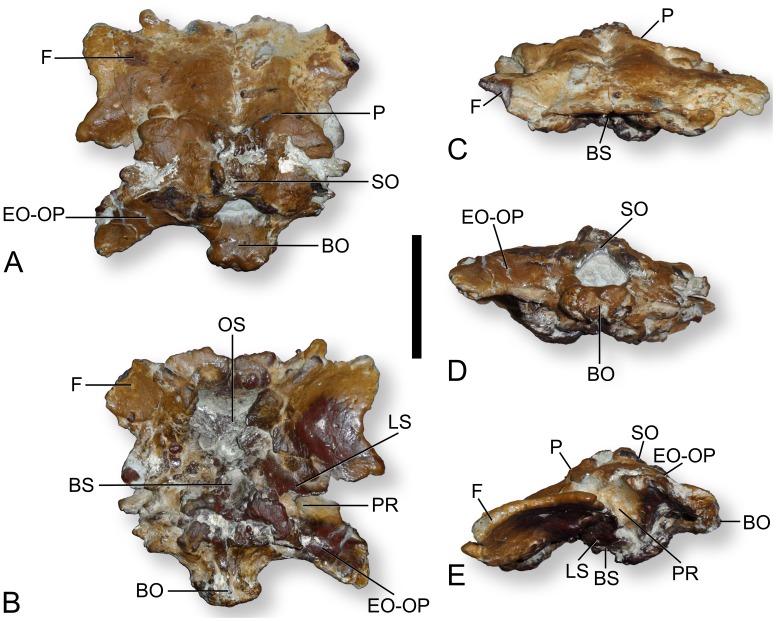
Photographs of the braincase of the titanosaurian sauropod *Ampelosaurus* sp. (MCCM-HUE-8741) from the Cretaceous of Fuentes, Spain. In dorsal (A), ventral (B), rostral (C), caudal (D), and left lateral (E) views. Abbreviations: BO, basioccipital; BS, basisphenoid; EO-OP, exoccipital-opisthotic/otoccipital; F, frontal; LS, laterosphenoid; OS, orbitosphenoid; P, parietal; PR, prootic; SO, supraoccipital. Scale bar equals 5 cm.

**Figure 2:**
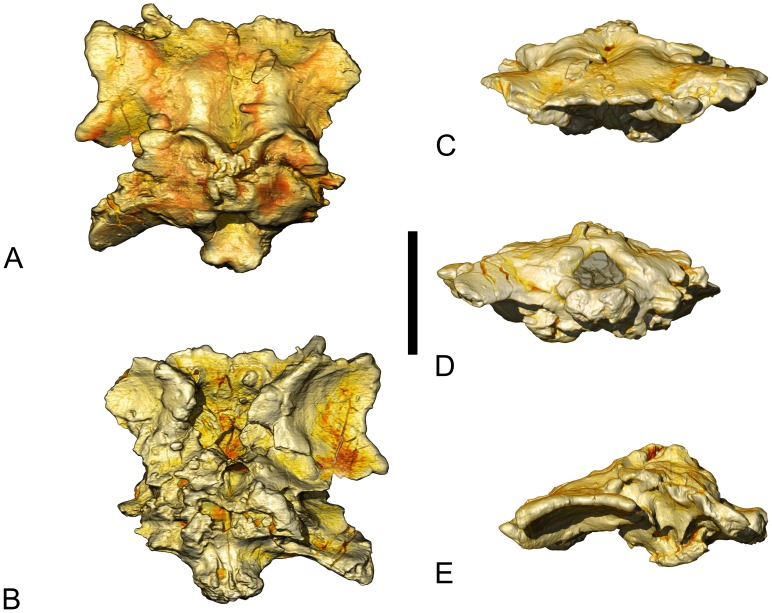
Volume-rendered CT-based images of the braincase of the titanosaurian sauropod *Ampelosaurus* sp. (MCCM-HUE-8741) from the Cretaceous of Fuentes, Spain. In dorsal (A), ventral (B), rostral (C), caudal (D), and left lateral (E) views. Scale bar equals 5 cm.

**Figure 3:**
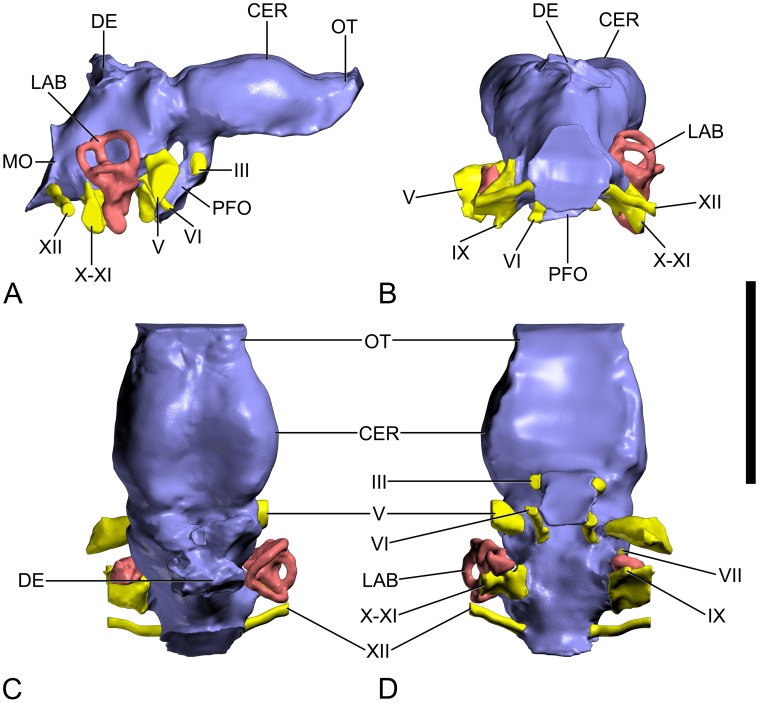
Surface-rendered CT-based images of the cranial endocast and endosseous labyrinth of the titanosaurian sauropod Ampelosaurus sp. (MCCM-HUE-8741) from the Cretaceous of Fuentes, Spain. In right lateral (A), caudal (B), dorsal (C), and ventral (D) views. Abbreviations: CER, cerebrum; DE, dural expansion; III, oculomotor nerve; IX, glossopharyngeal nerve; LAB, labyrinth; MO, medulla oblongata; OT, olfactory tract; PFO, pituitary fossa; V, trigeminal nerve; VI, abducens nerve; VII, facial nerve; X-XI, vagoaccessory nerve; XII, hypoglossal nerve. Scale bar equals 5 cm.

**Figure 4:**
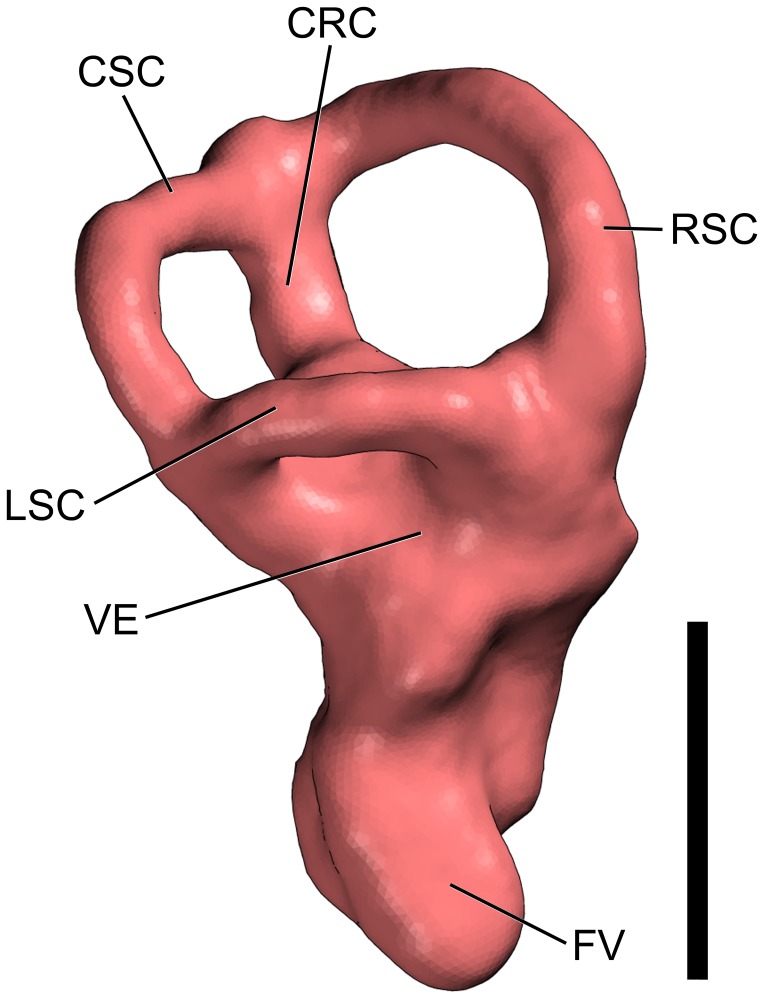
Surface-rendered CT-based image of the endosseous labyrinth of the right inner ear of the titanosaurian sauropod *Ampelosaurus* sp. (MCCM-HUE-8741) from the Cretaceous of Fuentes, Spain. In lateral view. Orientation was determined with the lateral semicircular canal held roughly horizontal. Abbreviations: CRC, crus commune; CSC, caudal ( = posterior, inferior) semicircular canal; FV, fenestra vestibuli ( = oval window); LSC, lateral ( = horizontal) semicircular canal; RSC, rostral ( = anterior, superior) semicircular canal; VE, vestibule of inner ear. Scale bar equals 1 cm.

The osteology of the specimen, MCCM-HUE-8741 (Fig. 1), will be contrasted with all the comparable Laurasian Late Cretaceous (Santonian-Maastrichtian) titanosaur specimens known so far. From Europe, these include the braincases of *Lirainosaurus astibiae* Sanz et al., 1999 [8] from Spain (MCNA 7439, [9]: figs. 2–4) and *Ampelosaurus atacis* Le Loeuff, 1995 [10] from France (MDE C3–761, [11]: fig. 4.2), as well as three braincases of indeterminate titanosaurians from France (Mechin private collection 225, [12]:***unnumb. pl.;*** ** ***MNHN unnumb.,*** [13]: fig. 2, pls 5–6; FAM 03.064, [14]) and another from Romania (FGGUB 1007, [15]: fig. 15, [16]: fig. 2.10). From Asia, these consist of the caudal portion of the skulls of *Nemegtosaurus mongoliensis* Nowinski, 1971 [17] (Z. PAL MgD-I/9, [6]: figs. 7–11, [17]: figs. 4–5, pls 12–13) and *Quaesitosaurus orientalis* Bannikov et Kurzanov *vide* Kurzanov et Bannikov, 1983 [2] (PIN 3906/2, [6]: fig. 18, [2]: fig. 2), both from Mongolia. Additional comparisons with taxa from areas south of the Tethys will also be made where pertinent.

Our knowledge of the paleoneuroanatomy of the Laurasian titanosauriforms rests on a single physical endocast of an indeterminate titanosauriform from the Early Cretaceous of Texas, USA (TMM 40435 [18]: fig. 2A). Our paleoneurological comparisons will, therefore, be extended to *Jainosaurus septentrionalis* (Huene et Matley, 1933) (GSI K27/497, [19]: fig. 6, [20]: fig. 7) from the Maastrichtian of India, as well as to further Gondwanan titanosaurians and even more remotely related taxa, when relevant.

To produce a three-dimensional reconstruction of the endocast of the cranial cavity and endosseous labyrinth of the inner ear, the specimen was scanned on a Yxlon CT Compact (Yxlon International, Hamburg, Germany) with a voltage of 180 kV and a current of 2.8 mA. The inter-slice spacing was of 0.20 mm. The in-plane pixel size was about 0.147 mm. The raw scan data were reconstructed using a bone algorithm. Data were output from the scanner in DICOM format and then imported into Avizo 7.0.1 (VSG, Burlington, MA, USA) for viewing, analysis, and visualization. The resulting 3D models were then imported into the 3D modeling software Maya 2012 (Autodesk, San Rafael, CA, USA) for artifact removal, final rendering, and generation of the illustrations. The 3D PDFs in the Supporting Information were generated by exporting the 3D models from Maya into Deep Exploration 5.5 (Right Hemisphere, San Ramon, CA, USA) and then Adobe Acrobat 9 Pro Extended (Adobe Systems Inc., San Jose, CA, USA). The data are archived at the Departamento de Paleobiología of the Museo Nacional de Ciencias Naturales-CSIC (Madrid, Spain) and at WitmerLab at Ohio University (Athens, OH, USA).

## Results

### Osteology

MCCM-HUE-8741 (Figures 1, 2, S1, S2, S3) was discovered in August 2007 in the lowest part of the fossiliferous succession (G1; see [1]). It is of overall small size (length in the median axis: 100.8 mm; maximal width of the left, best preserved lateral half: 64.3 mm). Almost no sutures are visible. This is probably due to the fact that this is a mature titanosaurian in which the bones have largely fused together. Difficulties in discriminating sutures are exacerbated by the iron oxides that have penetrated the bone, concealing the bony surface and forming hard concretions in places that cannot be removed without jeopardizing the integrity of the specimen. Portions of the ventral half of the braincase (i.e., a small part of the basioccipital and some of the basisphenoid-parasphenoid) are missing. As a result, structures such as the basipterygoid processes cannot be appraised in any manner. The orbitosphenoid, which perhaps was incompletely ossified, is poorly preserved and has sunk into the cranial cavity. Nevertheless, the specimen does not appear to have suffered significantly from taphonomic deformation, as demonstrated, for instance, by its unaltered bilateral symmetry.

#### Frontal

The left frontal is complete. The rostrolateral corner of the right frontal is missing, but the break suggests that this happened during the excavation. The lateral margin of the frontal is remarkably sinuous, with two processes: one rostrolaterally and the other more caudolaterally. The rostral border of the rostrolateral process shows a large groove, for the articulation of the prefrontal, whereas the caudolateral process possibly articulated with the postorbital. The rostral margin is also pointed (close to the central axis), although in a much more subtle way. The dorsal surface of each frontal is uneven: it is convex along the central axis in the caudal half and concave elsewhere except in the zone of the rostrolateral process, where it is approximately flat. The ventral side of the frontal is marked by a large hemispheric depression whose lateral margin shapes into the two above-mentioned processes, which together constitute the roof of the orbit. The rostromedial part of the ventral surface is separated from this depression by a strong crest that runs caudally from the rostrolateral zone toward the midline of the united frontals, separating the orbital and narial portions of the braincase. The left frontal is 57.3 mm long and 64.3 mm wide.

The frontals of MCCM-HUE-8741 differ greatly from those of the Transylvanian titanosaurian braincase FGGUB 1007 ([15]: fig. 15). In the latter specimen, the lateral margin of the frontal is roughly straight, whereas in the Lo Hueco specimen the participation of the frontal in the orbital margin distinctly stands out laterally. Also, in the Transylvanian specimen, the frontals are oriented strongly ventrally from the articulation with the parietal. The ventral orientation of the frontals in MCCM-HUE-8741 is less prominent and, in particular, its dorsal surface does not show any strong ventral curvature. Although closer in morphology, the frontal of MCCM-HUE-8741 is also distinct from that of the specimen from Fox-Amphoux FAM 03.064 ([14]: figs. 2–5). Specifically, the latter has a continuously convex rostral edge and a straight lateral margin. In contrast, the frontal of *Ampelosaurus atacis* ([11]: fig. 4.2) resembles that of the specimen from Lo Hueco. It shows, in particular, the extensive contribution to the roof of the orbit and its correlated hemispheric depression on the ventral surface. However, the frontal of this species is not identical to that of MCCM-HUE-8741. For instance, the caudal border of the orbital roof is much more ventral in *A. atacis* ([11]: fig. 4.2E) than in the specimen from Lo Hueco and its lateral margin is not embayed ([11]: fig. 4.2A–B). The frontal of MCCM-HUE-8741 is clearly distinct from that of *Nemegtosaurus mongoliensis* ([6]: fig. 7), which has a fairly flat dorsal surface and mostly convex lateral margin marked with discrete transverse wrinkles. The frontal of *N. mongoliensis* ([6]: fig. 7) also bears a rostromedial depression, which is absent in MCCM-HUE-8741. The latter concavity is also present in *Quaesitosaurus orientalis* [6].

#### Parietal

The dorsal margin of the conjoined parietals is marked by a ω-shaped crest. The midpoint of this prominence contacts the supraoccipital caudally, whereas laterally the parietal sends two occipital wings. These extensions are not fully preserved, but they would have bordered the upper temporal fenestrae caudally, at least in their medial half. The caudal border of each occipital wing would have constituted the dorsal margin of the post-temporal fenestrae, but there is no evidence of these openings, suggesting they were either absent or situated laterally to the occiput as preserved. An aperture of angular outline is visible on the midline of the cranial roof near the frontoparietal contact. Its position is consistent with its identification as a pineal foramen (but see below). As preserved, the parietal is 79.6 mm wide.

The parietals of MCCM-HUE-8741 are clearly different from the unfused ones of the juvenile titanosaurian braincase FGGUB 1007 ([15]: fig. 15). The latter are extremely unusual in bearing rostromedially low, rounded outgrowths. In contrast, the conjoined parietals of *Ampelosaurus atacis* ([11]: fig. 4.2A) look similar in morphology to that of MCCM-HUE-8741. Le Loeuff ([11]:119–120) noted similarities between the parietal of *A. atacis* and that of *Antarctosaurus wichmannianus*, which does resemble that of the specimen from Lo Hueco in its arcuate dorsal crest ([21]:pl. 28 fig. 2, [22]:pls 63, 64 fig. e). The parietal of MCCM-HUE-8741 is also very close to that of the specimen from Fox-Amphoux ([14]: figs. 2–5), which possibly bore a similarly shaped crest. The parietal of MCCM-HUE-8741 is distinct from that of *Nemegtosaurus mongoliensis* ([6]: fig. 7, [17]: fig. 4a, pl. 13 fig. 1), which bears a prominent dorsal crest that is not nearly as biarcuate. The conjoined parietals of *N. mongoliensis* also show a short, flat median suture ([6]: fig. 7).

#### Supraoccipital

The precise morphology of the supraoccipital cannot be ascertained due to imperfect preservation. It appears to have been strongly convex and may have born a median nuchal crest (for ligament insertion), at least in its more dorsal part. It is presently pierced by two irregular apertures dorsally in its suture with the parietal. The participation of the supraoccipital to the foramen magnum cannot be known for sure, but it did not exceed the most dorsal quarter to judge from the position of the proatlas facets, which are typically borne by the exoccipitals. The supraoccipital is only 10.1 mm deep (dorsoventrally) and 16.5 mm long (rostrocaudally).

The supraoccipital of MCCM-HUE-8741 resembles that of the fragmentary titanosaur braincase described by Le Loeuff et al. [12] which has a massive nuchal crest, though this is a fairly widely distributed character in dinosaurs in general and in sauropods in particular. A strong nuchal crest is also present in the supraoccipital of *Ampelosaurus atacis* ([11]: fig. 4.2D).

#### Otoccipital

There is no way to distinguish the exoccipital from the opisthotic, and they presumably are co-ossified into a single complex (otoccipital), as is typical in archosaurs. Each otoccipital no doubt makes up most of the lateral margin of the foramen magnum. It is marked by a small protuberance in its dorsomedial area: the facet for the proatlas articulation. This bulge distorts the edge of the foramen magnum and thereby gives the latter a pyriform outline (21.1×19.1 mm). Whereas the medial otoccipital is strongly convex, the paroccipital process has a rather flat occipital surface. The latter, which is fusiform in section, is oriented ventrally and slightly caudally. Its state of preservation distally does not allow affirming if it originally bore a non-articulating ventral processes as in many titanosaurs, such as *Rapetosaurus krausei* ([3]: fig. 19) and *Saltasaurus loricatus* ([22]: fig. 19). The proximoventral margin of the otoccipital bears a groove-like depression, whose ventral border is the tuberal crest (crista tuberalis). CT scan data suggest that this furrow accommodated the single hypoglossal nerve (XII) as it left the braincase. The tuberal crest is extremely sharp and prominent. CT data reveal that it overhangs proximally the jugular foramen (foramen jugulare), which formed the exit of the vagoaccessory nerves (X–XI), and that the oval window (fenestra ovalis = fenestra vestibuli) opens just rostral to the latter. The better-preserved left otoccipital is 49.4 mm wide.

Compared with that of MCCM-HUE-8741, the paroccipital process of the braincase presented by Le Loeuff et al. [12] is much stouter. Thus, it is much higher at its base than the foramen magnum is wide ([12]:***unnumb. pl.***), whereas the height of the paroccipital process of the specimen from Lo Hueco is roughly similar to the transverse diameter of the foramen magnum. The specimen described by Le Loeuff et al. [12] bears a small protuberance at about midheight of the exoccipital. This somewhat recalls that seen in MCCM-HUE-8741, though it is not situated on the margin of the foramen magnum but slightly more distally. Similar protuberances are also found in the Dongargaon specimen ([23]: fig. 2) from the Maastrichtian of India and that from Balochistan ([24]: fig. 2C), Maastrichtian of Pakistan, as well as in other sauropods (e.g., *Spinophorosaurus nigerensis* [25]: fig. 3B, S1, S2, S3). In contrast, no such feature is seen on the otoccipital of *Lirainosaurus astibiae* ([9]: fig. 2A), *Ampelosaurus atacis* ([11]: fig. 4.2D), and the specimen reported by Allain ([13]:pl. 6 fig. 1A). The paroccipital process of the latter specimen is oriented also more strongly caudally than that of the Lo Hueco specimen. The otoccipital of MCCM-HUE-8741 is clearly distinct from that of *Nemegtosaurus mongoliensis* ([6]: fig. 7, [17]: fig. 5a), whose paroccipital process is proportionally higher (dorsoventrally) and more ventrally inclined. In addition, the otoccipital of *N. mongoliensis* ([6]: fig. 7) does not bear any noticeable proatlas facet. It displays, however, a prominent ridge that extends from the dorsolateral margin of the foramen magnum onto the paroccipital process, but subsides at the midlength of it. A comparable elongate prominence is present in *Quaesitosaurus orientalis* ([6]: fig. 18), whose paroccipital process has a tapered prong distoventrally ([6]: fig. 18). In both *N. mongoliensis* and *Q. orientalis*, the foramen magnum is oval with the long axis oriented dorsoventrally ([2]: fig. 2a, [6]: figs. 9, 18, [17]: fig. 5a, pl. 12 fig. 2).

#### Basioccipital

The basioccipital of the specimen from Lo Hueco is remarkable in having an occipital condyle that is much broader laterally than high in caudal view. Thus, the occipital condyle is wide (a little wider than the foramen magnum), but it is dorsoventrally low. The non-hemispherical form of the occipital condyle, which appears to be genuine given the lack of any indication of effective taphonomic compression, might have favored the dorsoventral and mediolateral motions of the head over those in the diagonal. The lowness of the condyle is responsible for a condylar neck whose lateral surfaces are very convex dorsoventrally, whereas the ventral side is much flatter. Therefore, the occipital condyle is not well separated from its neck ventrally. However, and despite the wideness of the neck, the occipital condyle stands out from it laterally (best seen on the better-preserved left size). The irregular surface of the occipital condyle is at least partly related to the loss of the original cartilaginous covering. No participation of the otoccipital in the occipital condyle is evident, but almost all sutures are obliterated. A part of the left basal tuber is visible at this level, which means that the complete braincase was especially low (the skull as a whole may have been high, though). Probably only the dorsal half or so of the preserved basal tuber was made up by the basioccipital. It is oriented laterally, and its lateral surface appears to have been rugose, which is common in titanosaurians. The occipital condyle is 28.6 mm wide and 15.8 mm deep. The tubera:condyle width ratio of MCCM-HUE-8741 is very high (at least 2.33).

Like MCCM-HUE-8741, *Ampelosaurus atacis* has a transversely ovoid occipital condyle ([11]: fig. 4.2D). The juvenile titanosaurian braincase FGGUB 1007 ([15]: fig. 15) also has a somewhat laterally elongate occipital condyle, but it appears more rounded ventrally. The basioccipital of *Lirainosaurus astibiae* ([9]: figs. 2–3) is clearly different from that of the specimen from Lo Hueco. For instance, the occipital condyle is much taller (more hemispherical) in *L. astibiae* than in MCCM-HUE-8741. The ventral border of the articular surface of the occipital condyle stands out from the neck in a very marked way in *L. astibiae*, whereas this border is almost in continuity with the neck in the specimen from Lo Hueco. In addition, the basal tubera of *L. astibiae* are significantly more ventrally extended than those of the specimen from Lo Hueco, hinting at a deeper braincase in the former. They are also positioned much more caudally, more or less at the vertical level of the occipital condyle, whereas they are approximately at the vertical level of the supraoccipital in MCCM-HUE-8741. The basioccipital of the titanosaurian braincase reported by Allain [13] is also clearly different from that of MCCM-HUE-8741. For instance, in that specimen the occipital condyle is a bit deformed, but subtriangular in outline, not ovoid as in the Lo Hueco specimen. Its neck is also a little longer in proportion, the articular surface of the condyle extends ventrally beyond the ventral border of the neck and, above all, the ventrolateral sides of the neck are concave. The basal tubera have a more habitual (more ventral) position. In fact, the basal tubera emerge at a variable level in sauropods. Thus, the camarasaurid *Camarasaurus lentus* ([26]: fig. 23A), the dicraeosaurid *Dicraeosaurus hansemanni* ([27]: fig. 96), and other species (including most titanosaurians) have more ventral basal tubera than does MCCM-HUE-8741, which is similar in this respect to some species such as the brachiosaurid *Giraffatitan brancai* ([27]: figs. 5, 7), two other basal titanosauriforms ([18]: fig. 1B, D, [28]: fig. 3A, D), and the titanosaurians *Mongolosaurus haplodon* ([29]: fig. 2A) and *Tapuiasaurus macedoi* ([7]: fig. 1D). In MCCM-HUE-8741, the neck of the occipital condyle does not show the possibly autapomorphic ventral groove described by Díez Díaz et al. [14] in FAM 03.064. The basioccipital of MCCM-HUE-8741 differs unmistakably from that of *Nemegtosaurus mongoliensis* in the relative size and the outline of the occipital condyle. Thus, in *N. mongoliensis*, the occipital condyle is more than twice as broad as the foramen magnum and its dorsal surface is distinctly concave in the midline, whereas the ventral surface is convex in caudal view ([6]: fig. 9, [17]: fig. 5a, pl. 12 fig. 2). Whereas the difference in relative size of the occipital condyle between MCCM-HUE-8741 and *Quaesitosaurus orientalis* is less marked than it is between the former and *N. mongoliensis*, the difference in shape is more marked, as *Q. orientalis* has a fairly hemispheric occipital condyle ([2]: fig.2, [6]: fig. 18). The condyle width:height ratio of MCCM-HUE-8741 (1.81) are only approximated among sauropods by that of *A. atacis* (1.79) and that of an indeterminate titanosaurian braincase (1.86) described by Paulina-Carabajal and Salgado ([30]: fig. 2D), whereas the tubera:condyle width ratios (which cannot be ascertained in MDE C3–761 ) is close to that of the titanosaurian *Bonatitan reigi* (2.24) ([29]:tab. 1).

#### Basiphenoid-parasphenoid

Based on CT scan data, this bone complex (parabasisphenoid) would constitute the floor of the preserved braincase from the rostral edge of the basioccipital to the rostral wall of the pituitary fossa. The ventral half or so of the preserved basal tuber is probably made up by the basisphenoid. The pituitary fossa is ovoid in section, wider than long, and the dorsum sellae is caudoventrally inclined. Very close to the pituitary fossa, on both sides of it, the basisphenoid is pierced by a small foramen caudally. CT scan data substantiate that it was for the abducens nerve (VI), which therefore did not enter the pituitary fossa in contrast to what is generally observed in most sauropods [31]. In its middle part, the basisphenoid-parasphenoid is about 40.3 mm wide. The section of the pituitary fossa is 6.9 mm wide and 3.7 mm long.

As in MCCM-HUE-8741 and most sauropods, the pituitary fossa seems to have been inclined caudoventrally in *Ampelosaurus atacis* ([11]: fig. 4.2) and *Lirainosaurus astibiae* ([9]: figs. 2–4). This was also the case in the specimen reported by Allain [13], to judge from the strongly caudoventrally oriented basisphenoid-parasphenoid.

#### Orbitosphenoid

The orbitosphenoid, which is rostral to the pituitary fossa and laterosphenoid, is poorly preserved and displaced dorsally. The left orbitosphenoid better preserves the border of a single median aperture for the optic nerve (II). The orbitosphenoid also ventrally borders the origin of the olfactory tracts (I), by flooring the wide rostral aperture of the braincase. The remaining border of the olfactory tract aperture (laterally and dorsally) was constituted by the frontals. As preserved, the orbitosphenoid extends rostrally from the pituitary fossa 17.5 mm along its median axis.

The original configuration of the orbitosphenoid of MCCM-HUE-8741 was no doubt similar to that seen in *Ampelosaurus atacis* ([11]: fig. 4.2B–C), with a single conjoined optic foramen and a broad, but low, olfactory aperture. A single optic foramen seems to be homoplastically distributed in sauropods as it also appears to be present in the basal eusauropod *Shunosaurus lii* ([32]: fig. 7B) and *Giraffatitan brancai* ([27]: figs. 5–6), whereas *Dicraeosaurus hansemanni* ([27]: figs. 136–137), *Camarasaurus lentus* ([26]: fig. 24A), and *Nemegtosaurus mongoliensis* ([6]: fig. 8) have two distinct openings.

#### Prootic

The prootic is a dorsoventrally high but rostrocaudally short bone. It is bordered largely by the basisphenoid ventrally, the laterosphenoid rostrally, the parietal dorsally, and the otoccipital caudally. The prootic is marked by a robust and sharp otosphenoidal crest (crista otosphenoidalis = crista prootica), which runs rostroventrally from the basis of the paroccipital process to contact the basal tuber and most probably continued ventrally to reach the basipterygoid process. This crest borders caudally the large trigeminal foramen. The rostrocaudal distance between the otosphenoidal crest and the antotic crest (crista antotica) of the laterosphenoid (which corresponds approximately with the caudal and rostral limits of the prootic) is 10.6 mm at the level of the trigeminal foramen.

The otosphenoidal crest of MCCM-HUE-8741 is sharper and more prominent than that of *Ampelosaurus atacis* ([11]: fig. 4.2B, E). This difference, however, is more marked with *Lirainosaurus astibiae* ([9]: figs. 3A, 4C), the specimen reported by Allain [13], and FAM 03.064 ([14]: figs. 4–5).

#### Laterosphenoid

The laterosphenoid of MCCM-HUE-8741 is well preserved on the left side. It is bordered ventrally by the basisphenoid, rostrally by the orbitosphenoid, dorsally by the frontal and the parietal, and caudally by the prootic. The laterosphenoid is characterized by a lamellar and slightly arcuate capitate process that projects laterally and fits along the caudolateral border of the frontal. In so doing, it participates to the separation of the adductor chamber from the orbital cavity. The ventromedial continuation of the capitate process, the antotic crest, apparently constituted most of the rostral margin of a large, somewhat heart-shaped pit at the bottom of which opens the trigeminal foramen. Rostrally to the latter, on the suture with the basiphenoid, a small foramen provided exit to the oculomotor (III) and, possibly, the trochlear nerves (IV), too. The left laterosphenoid is 33.3 mm wide and 19.8 mm long.

The comparisons of the laterosphenoid of MCCM-HUE-8741 with that of the other Laurasian Late Cretaceous titanosaurian braincases, in which this element is either lacking or fused indistinctly with surrounding bones like the orbitosphenoid, are difficult or impossible. However, MCCM-HUE-8741 appears in this respect much closer to *Ampelosaurus atacis* ([11]: fig. 4.2B) than to the specimen reported by Allain [13], which presents a stronger capitate process.

### Neuroanatomy

The first virtual cranial cavity endocast of a dinosaur (a theropod) was generated from CT scans more than a decade ago [33]–[34]. This method was first applied to a sauropod some years later ([35]: fig. 25), nearly a century after the first published detailed figuration of a physical endocast made from a specimen of this group ([36]: fig. 16A). In the present case, the processing of the CT data resulted in a very close rendering of the cranial endocast and endosseous labyrinth (Figures 3, 4, S1, S2, S3). Due to the imperfect preservation of the braincase (displacement of the orbitosphenoids into the cranial cavity, etc.), the rostroventral part of the endocast is missing. As a consequence, the exact position and configuration of the optic (II) nerve could not be determined. For the sake of ease of description, we will refer to the reconstructed digital casts of bone-bounded spaces that housed soft-tissue structures as if they were the structures themselves (e.g., “trigeminal nerve” instead of “digital cast of trigeminal canal”).

#### Brain

As a consequence of the dorsoventral compression of the braincase, the endocast has moderate pontine and cerebral flexures (about 40°). As in *Jainosaurus septentrionalis* ([19]: fig. 6, [20]: fig. 7) and most other non-avian archosaurs, the hindbrain and midbrain are relatively poorly outlined in the endocast due to former meningeal coverings and apparently substantial dural venous sinuses, which obscure details of the optic lobes and the cerebellum. In contrast with TMM 40435 ([18]: fig. 2A) and a few other taxa such as cf. *Cetiosaurus oxoniensis* ([37]: fig. 6) and *Giraffatitan brancai* ([27]:pl. 13 figs. 1–2), no “nub” betraying the location of the cerebellum can be discerned. As in TMM 40435 [18] and many other archosaurs, the hindbrain is especially narrow at the level of the otic capsules in MCCM-HUE-8741.

The cerebral region is separated from the hindbrain-midbrain complex through a marked constriction caused by the laterosphenoid pillar in the endocranial cavity. In contrast with the subset caudal to this pillar, the cerebral region of the brain is also relatively well defined. In fact, it forms two reniform swellings in dorsal view. The concavity of each one faces medially, resulting in a median depression, a little off-centered caudally. We interpret this morphology as being due to paired longitudinal dural venous sinuses that coursed dorsolaterally through the cerebral region, as previously described for *Diplodocus longus* ([31]: fig. 6.9D). The cleft between these pronounced venous swellings is very deep and broad suggesting that the two cerebral hemispheres were very little inflated and must have been extremely modest. This configuration is markedly different from that seen in the other few sauropods with thin dural covering of the cerebral regions. Thus, in the rebbachisaurid *Nigersaurus taqueti* ([38]: figs. 1F, S4) and the titanosaurians *Antarctosaurus wichmannianus* ([39]: fig. 5B) and *Bonatitan reigi* ([39]: fig. 2B), there is no median longitudinal indentation in the cerebral region. This is especially worth mentioning for the latter two taxa because their braincase is not fundamentally different in overall morphology from MCCM-HUE-8741 ([22]:pls 62–63, [40]: figs. 7–8). Rostrally, the cerebral region prolongs into the olfactory tracts, whose dorsal aspect could be reconstructed as it was roofed by the frontal. The caudalmost part of the cerebral region of the Spanish specimen is topped by only a moderate dural expansion. This is at odds with *Jainosaurus septentrionalis* ([19]: fig. 6, [20]: fig. 7) in which this venous feature is responsible for a sharp process on the endocast, about dorsal to the putative location of the optic lobes. However, relatively much more substantial dural expansions are known in the diplodocoid sauropods *Dicraeosaurus hansemanni* ([27]:pl. 13 figs. 6–7) and *Diplodocus longus* ([31]: fig. 6.9). In MCCM-HUE-8741, the small median opening in the skull roof near the frontoparietal contact is responsible for a swelling on the endocast that is evocative of a pineal system. It is in the exact position where the pineal gland is expected to have evaginated, between the forebrain and the midbrain. However, the state of the bony margins of the aperture in MCCM-HUE-8741 calls its genuineness and its identification as a pineal foramen into question. Actually, the bone roofing this region in sauropods is commonly very thin and damaged (see discussion about the “pineal hypothesis” in sauropods in [31]:pp. 79–80). The pituitary fossa is incomplete distally, but was directed strongly caudally as indicated by the sturdily inclined dorsum sellae. This is reminiscent of the condition in the titanosaurian *Bonatitan reigi* ([39]: figs. 1–3) and many other archosaurs.

In striking contrast with more basal sauropods such as *Spinophorosaurus nigerensis* ([25]: fig. 4, S1, S2, S3), there is no evidence of a complex endocranial venous system that is highly anastomotic and partly invades the laterosphenoid laterally and the occiput caudally, but this absence is possibly due to issues pertaining to both preservation and the resulting quality of the CT scan data. According to Kurzanov and Bannikov ([2]:p. 95), in *Quaesitosaurus orientalis* the middle cerebral vein is accommodated by an arcuate groove on the inner surface of the braincase before passing through the wall of it. This configuration is reminiscent of a variety of sauropods (e.g., cf. *Cetiosaurus oxoniensis* ([37]: fig. 6), *Dicraeosaurus hansemanni* ([27]:pl. 13 fig. 6–7), *Giraffatitan brancai* ([41]: figs. 1–2) and indeed many other archosaurs, but not any titanosaurian (see e.g., [39]), and thus its presence in *Q. orientalis* merits confirmation.

#### Cranial nerves

A single nerve trunk emerges from the lateral side of the hypophyseal peduncle. Given the displacement of the orbitosphenoid in MCCM-HUE-8741, it is difficult to determine whether this single aperture is just for the oculomotor (III) nerve or potentially also for the trochlear (IV) nerve. Both conditions are found among sauropods. For example, in *Jainosaurus septentrionalis* ([19]: fig. 6, [20]: fig. 7), *Camarasaurus lentus* ([31]: fig. 6.8), and many other sauropods, these two nerves are more or less close to one another, but not fused. However, this combined oculomotor-trochlear aperture does occur in taxa such as *Nigersaurus taqueti* ([38]: fig. 1, S4), *Suuwassea emilieae* (specimen ANS 21122), *Dicraeosaurus hansemanni* ([27]:pl.13 figs. 6–7), and *Diplodocus longus* ([31]: fig. 6.9). It remains a possibility that the trochlear nerve emerges more dorsally, between the laterosphenoid and orbitosphenoid, as seen in other taxa.

More caudally on the brainstem, the trigeminal nerve (V) comes into view. As usual in archosaurs, it is the largest of the cranial nerves. The roughly heart-shaped outline of the trigeminal foramen is related to the division of this nerve into rostral (ophthalmic, V_1_) and caudal (maxillomandibular, V_2,3_) rami. In *Jainosaurus septentrionalis* ([19]: fig. 6, [20]: fig. 7), the trigeminal foramen presents a very unusual slit-like internal outline.

The abducens nerve (VI) emerges from the ventral surface of the brainstem, courses rostroventrally and, as far as it can be observed, passes lateral to the pituitary fossa. This is in contrast with the general condition in sauropods (see e.g., cf. *Cetiosaurus oxoniensis* ([37]: fig. 6), *Diplodocus longus* ([31]: fig. 6.9), *Giraffatitan brancai* ([41]: fig. 1)) in which the abducens nerve enters the pituitary space. The same primitive pattern is seen in TMM 40435 ([18]: fig. 2A), but not in titanosaurians [39], and thus MCCM-HUE-8741 conforms to the derived condition observed in this clade.

The facial nerve (VII) emerges caudal to the trigeminal nerve (V) and passes ventrolaterally. The situation of the facial nerve with respect to the trigeminal nerve is identical in MCCM-HUE-8741 and *Bonatitan reigi* ([39]: fig.2). In other taxa, the facial nerve may be closer to the trigeminal nerve (e.g., cf. *Cetiosaurus oxoniensis* ([37]: fig. 6) or in the vicinity of the otic region (e.g., *Camarasaurus lentus* ([31]: fig. 6.8)). As in other sauropods and reptiles in general (see [42]), the facial nerve of MCCM-HUE-8741 is of very small diameter.

A great deal of caution needs to be exercised in inferring the course of the glossopharyngeal nerve (IX). Our tentative interpretation of the CT scan data is that this nerve emerges from the brain stem and penetrates the braincase wall together with the vagoaccesory nerve (X-XI) and the internal jugular vein through a dorsoventrally elongated foramen in (or mostly constituted by) the otoccipital, the metotic fissure (fissura metotica). However, it possibly left the braincase through a small aperture of its own, the occipital ***recess*** (recessus scalae tympani), which is located at the ventral end of the otoccipital, about where the tuberal crest subsides. Hence, MCCM-HUE-8741 might shows an unusual configuration in which the glossopharyngeal nerve appears to be separate from and ventral to the vagoaccesory nerve, in contrast to the condition known in other sauropods (see e.g., [20], [25], [31], [39], [41], [43]), in which the glossopharyngeal and vagoaccesory nerves are more closely arranged.

The vagoaccesory nerve (X-XI) leaves the braincase through a moderately-sized jugular foramen that is located immediately caudally to the oval window. Tidwell and Carpenter ([18]: fig. 2A) identified an independent accessory nerve (XI) at mid-distance between the fissura metotica/jugular foramen and a single hypoglossal foramen. This is most probably incorrect as this stands out in sharp contrast with the general condition, in which the accessory nerve (XI) has joined the vagus (X) (see e.g., [44]).

The hypoglossal nerve (XII) has a single root entering the braincase wall (otoccipital) very close to the rim of the foramen magnum. This situation suggests it is homologous with the caudalmost hypoglossal root of other sauropods. Such a configuration is highly remarkable because most sauropods, other than titanosaurians (including *Jainosaurus septentrionalis* ([20]: figs. 4, 7)), have two hypoglossal roots ([31], [39]). The basal titanosauriform *Giraffatitan brancai* has also two roots ([27]: fig. 116). We interpret the putative accessory nerve of TMM 40435 ([18]: fig. 2A) as actually a rostral root of the hypoglossal nerve, which suggests that this specimen is from a primitive, non-titanosaurian, titanosauriform.

#### Inner ear

A beautiful rendering of the right labyrinth could be arrived at, whereas merely the vestibule and base of the lagena were reconstructed on the left side. The semicircular canals are contracted, i.e. the radius of the arc they describe is small and, therefore, they are highly curved. The longest semicircular canal (but only moderately so) is the rostral one and the lateral semicircular canal is the shortest, which is the most common configuration in vertebrates [45]–[46]. The semicircular system of MCCM-HUE-8741 shows also a plesiomorphic morphology in that the planes delimited by each semicircular canal do not intersect; that is, neither of the vertical semicircular canals passes ventral to the lateral one and not any of the former extends behind or ahead of their common leg (crus commune) [47]. The latter presents a blunt apex. Another archaic character is that the ampullae of the semicircular canals are not discernibly inflated, which is to weigh against the sizeable vestibule. The rostral semicircular canal is oriented at an angle of about 45° with the midline of the endocast. The angle between the rostral and lateral semicircular canals is about 85° (85° in *Spinophorosaurus nigerensis*, 100° in *Giraffatitan brancai*), that between the two vertical semicircular canals is about 75° (75° in *S. nigerensis*, 80° in *G. brancai*), and that between the posterior and lateral semicircular canals is about 95° (100° in *S. nigerensis*, 95° in *G. brancai*). The lagena (cochlear duct) is extremely short, not extending ventrally beyond the oval window. The latter draws indeed a dorsoventrally elongate oval.

The labyrinth of MCCM-HUE-8741 is largely reminiscent of that of other titanosaurians such as *Bonatitan reigi* and *Antarctosaurus wichmannianus* ([39]: fig. 9). It is markedly different from that of the basal sauropod *Spinophorosaurus nigerensis* ([25]: fig. 5, S1, S2, S3). More surprisingly, the labyrinth of MCCM-HUE-8741 is equally distinct from that of the basal titanosauriform *Giraffatitan brancai* ([27]: figs. 119–126). These differences, which rest in part on the significantly more elongate semicircular canals in *S. nigerensis* and *G. brancai*, are discussed below.

## Discussion

The specimen from Lo Hueco is especially remarkable in its dorsoventral compression, most of which seems to be natural, although there is little doubt that some deformation occurred postmortem. Dorsoventral compression of the braincase is an uncommon character in sauropods (and saurischians as a whole), but it is shared with the titanosaurian braincases of the *Isisaurus*-morph *sensu* Wilson et al. [20]. This is in contrast with other European Late Cretaceous titanosaurian braincases, such as FGGUB 1007 ([15]: fig. 15), which is short and deep. The titanosaurian braincases of the *Isisaurus*-morph also resemble MCCM-HUE-8741 in having an occipital condyle that is strongly deflected ventrally relative to the skull roof (frontals and parietals) so that the long axis of the occipital condyle approximates the plane of the occiput (paroccipital processes and supraoccipital). In contrast, the long axis of the occipital condyle and the plane of the skull roof are subparallel in taxa such as *Nemegtosaurus mongoliensis* [6].

Although the number of sauropod braincases from the Late Cretaceous European archipelago found to date is limited, it shows a significant diversity. As evidenced by the comparisons above, the specimen from Lo Hueco resembles the braincase of *Ampelosaurus atacis* MDE C3–761 ([11]: fig. 4.2). This last specimen is yet to be described in detail but, as far as these two braincases can be compared, they are fairly similar. Naturally, they present a number of characters that are more or less widely distributed in titanosaurians including fused frontals, a frontal with a medial convexity on the dorsal surface, a contribution of the frontal to the margin of the upper temporal fenestra, a rostrocaudally short upper temporal fenestra (and therefore weak jaw adductors) that faces dorsolaterally, a rostral portion of parietal that is relatively broad and a caudal portion that is inclined caudally with a marked dorsal crest and laterally extended occipital wings, and other features. But, more significantly, both MCCM-HUE-8741 and MDE C3–761 have a flat occiput with transversely oriented paroccipital processes. According to Curry Rogers ([48]:appx 2.1), such an occipital plate is known only in one other titanosaurian in her character/taxon matrix: *Jainosaurus septentrionalis*. However, MCCM-HUE-8741 is easily distinguished from *J. septentrionalis*, whose occipital condyle is hemispheric and with a long axis subparallel to the skull roof [20]. MCCM-HUE-8741 therefore appears closer in morphology to *A. atacis* than to any other titanosaurian, especially any other specimens from the Santonian-Maastrichtian from north of the Tethys known so far. The sizes of MCCM-HUE-8741 and MDE C3–761 are also commensurate.

However, there appear also to be differences between MCCM-HUE-8741 and MDE C3–761. Thus, the latter is less dorsoventrally compressed and lacks proatlas facets. Also, the frontal orbital margin of MDE C3–761 is not embayed, whereas there is a rostrolateral and a caudolateral prong on the frontal in MCCM-HUE-8741. In view of the above-mentioned similarities, we judge that a provisional attribution of MCCM-HUE-8741 to the genus *Ampelosaurus* is reasonable. However, we find it difficult to assess the significance of the differences between MCCM-HUE-8741 and MDE C3–761 given our extremely poor knowledge of intraspecific variation in cranial morphology among sauropods in general and titanosaurians in particular. Hence, the former is better left at this time in open nomenclature as *Ampelosaurus* sp. Incidentally, strongly spatulate teeth alike those of *A. atacis* were discovered at Lo Hueco ([1]:p. 1275). The presence in mid-eastern Spain of a sauropod species close (or identical) to one from approximately contemporaneous sediments in south-western France (at present almost 500 km farther northeast) is actually not unexpected. The Iberian Massif island (on which Lo Hueco is situated) and the emerged area of the Ebro Massif (where Bellevue, the type locality of *A. atacis*, is located) were admittedly separated by a shallow strait in early Campanian time [49]. However, this seaway had disappeared by the late Maastrichtian due to marine retreats, removing a serious barrier to the interchange of large land vertebrates between the Iberian Massif and the French Craton through the Ebro Massif and the Corsica-Sardinia Block [50].

From a paleoneuroanatomical viewpoint and if we take our reconstruction (which is unavoidably a simplified and somewhat distorted reflection of the genuine neuroanatomy) at face value, the endocast of MCCM-HUE-8741 is as dissimilar from that of a more basal form such as *Spinophorosaurus nigerensis* [25] as it is reminiscent of other titanosaurians [39]. The cerebral region appears to have been covered by a tight dural envelope that accommodated a potentially simple venous system, for the most part reduced to longitudinal sinuses. This results in an “unadorned” and straightforwardly interpretable endocast (as far as the cerebral region is concerned at least). In addition, even though the cranial nerves present roughly the configuration seen in other sauropods, there are derived features that appear to characterize titanosaurians, such as the abducens nerve (VI) that passes lateral to the pituitary fossa rather than entering it and the hypoglossal nerve (XII) that exits the skull via a single foramen [39]. These features are at variance with most saurischians, including *Giraffatitan brancai*, which is a basal titanosauriform [41]. In contrast with *Spinophorosaurus nigerensis* and *Giraffatitan brancai* but similar to other sauropods such as *Camarasaurus lentus* ([38]: S5F–G), the labyrinth shows a dramatic reduction of the magnitude of the vestibular system such that the rostral semicircular canal is not much longer than the caudal canal.

The functional significance of the differential development of the semicircular canals in sauropods has been recently brought up by Knoll et al. [25]. It has long been hypothesised (see e.g., [51]:pp. 19, 161) that the greater the radius of the semicircular canals, the greater the locomotor agility of its holder. Again recently, Cox and Jeffery [52] showed that the canal radii were positively correlated with agility of locomotion in mammals. The same authors [53] demonstrated that the semicircular canal morphology in some mammals is arranged primarily for perceiving head movements and then secondarily for sending their impulses for compensatory eye movements (vestibulo-ocular reflex). A number of reports have suggested relationships between semicircular canal morphology and locomotor agility in birds as well. For instance, Hadžiselimović and Savković [54] have demonstrated that the canals are short and thick with poorly marked ampullae in clumsy fliers. In contrast, few investigations have focused on the possible correlation between morphology of the semicircular canals and behavioral pattern in reptiles (see in particular [55]). Inasmuch as no sauropod would qualify as a physically nimble animal, the development of the semicircular canals in any given taxon may be more probably related, inter alia, with the natural range of turning movements of its head.

## Supporting Information

Figure S1 **Interactive visualization made from the CT scan of the braincase of the sauropod dinosaur** ***Ampelosaurus*** **sp. (MCCM-HUE-8741) from the Late Cretaceous of Fuentes, Spain (small file).**
(PDF)

Figure S2 **Interactive visualization made from the CT scan of the braincase of the sauropod dinosaur** ***Ampelosaurus*** **sp. (MCCM-HUE-8741) from the Late Cretaceous of Fuentes, Spain (medium file).**
(PDF)

Figure S3 **Interactive visualization made from the CT scan of the braincase of the sauropod dinosaur** ***Ampelosaurus*** **sp. (MCCM-HUE-8741) from the Late Cretaceous of Fuentes, Spain (large file).**
(PDF)
