# Supplementary figures and images for: Neurocranial Osteology and Neuroanatomy of a Late Cretaceous Titanosaurian Sauropod from Spain (Ampelosaurus sp.)
Source: PLoS One. 2013 Jan 23;8(1):e54991. doi: 10.1371/journal.pone.0054991 (PMC3552955; doi:10.1371/journal.pone.0054991)

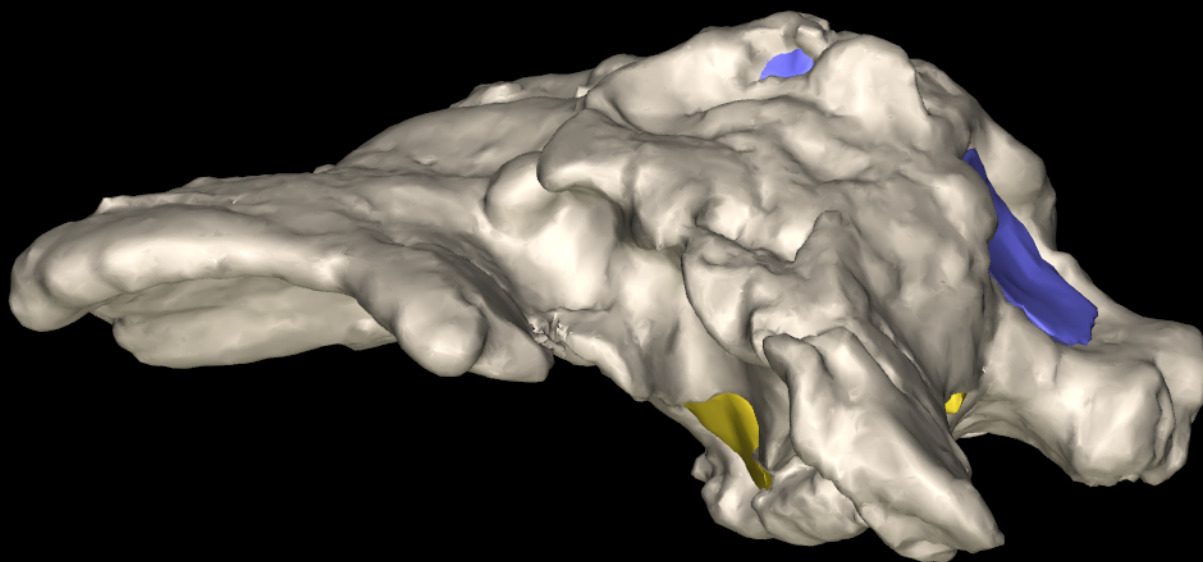

Supplement: Figure S1 — Interactive visualization made from the CT scan of the braincase of the sauropod dinosaur Ampelosaurus sp. (MCCM-HUE-8741) from the Late Cretaceous of Fuentes, Spain (small file). (PDF) [file pone.0054991.s001.pdf]

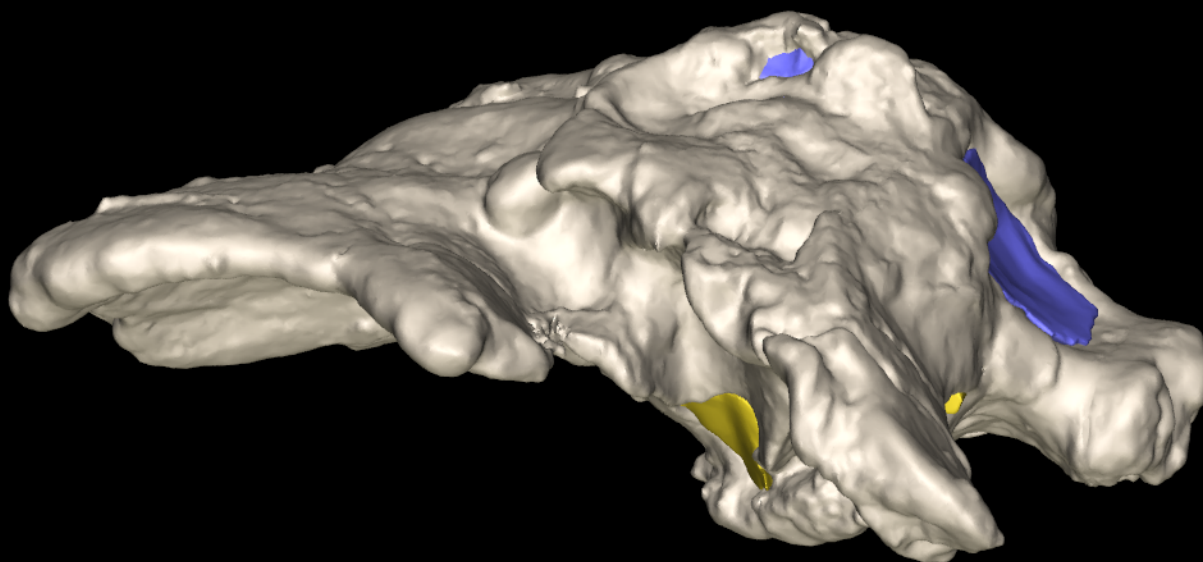

Supplement: Figure S2 — Interactive visualization made from the CT scan of the braincase of the sauropod dinosaur Ampelosaurus sp. (MCCM-HUE-8741) from the Late Cretaceous of Fuentes, Spain (medium file). (PDF) [file pone.0054991.s002.pdf]

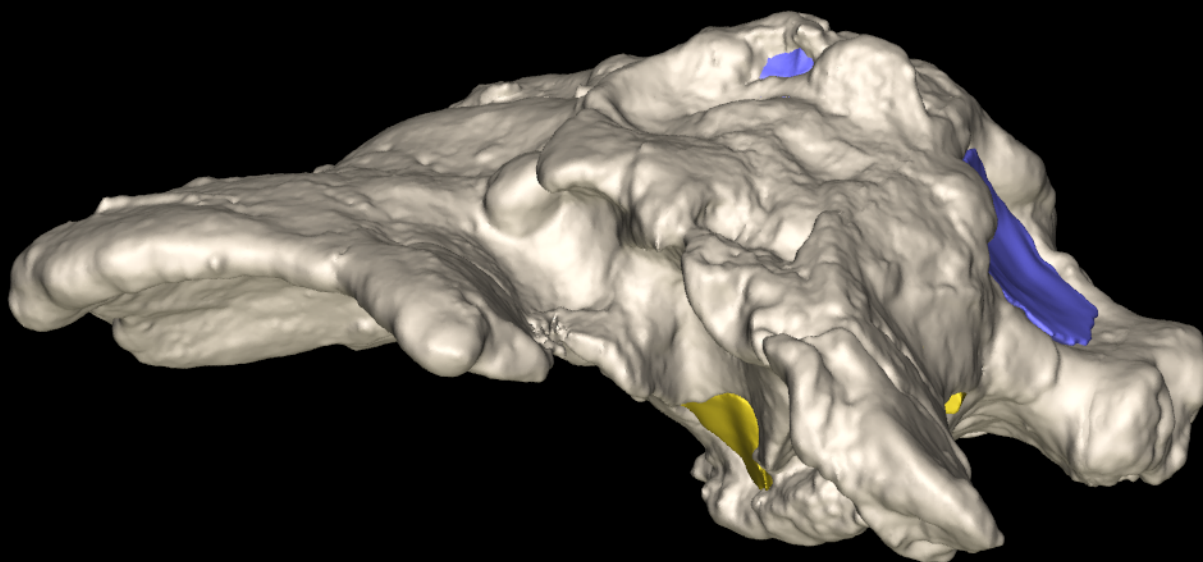

Supplement: Figure S3 — Interactive visualization made from the CT scan of the braincase of the sauropod dinosaur Ampelosaurus sp. (MCCM-HUE-8741) from the Late Cretaceous of Fuentes, Spain (large file). (PDF) [file pone.0054991.s003.pdf]
